# Mapping the in vivo fitness landscape of a tethered ribosome

**DOI:** 10.1126/sciadv.ade8934

**Published:** 2023-04-28

**Authors:** Felix Radford, Jesse Rinehart, Farren J. Isaacs

**Affiliations:** ^1^ Department of Molecular, Cellular, and Developmental Biology, Yale University, New Haven, CT 06520, USA.; ^2^ Systems Biology Institute, Yale University, West Haven, CT 06516, USA.; ^3^ Department of Cellular and Molecular Physiology, Yale School of Medicine, New Haven, CT 06520, USA.; ^4^ Department of Biomedical Engineering, Yale University, New Haven, CT 06520, USA.

## Abstract

Fitness landscapes are models of the sequence space of a genetic element that map how each sequence corresponds to its activity and can be used to guide laboratory evolution. The ribosome is a macromolecular machine that is essential for protein synthesis in all organisms. Because of the prevalence of dominant lethal mutations, a comprehensive fitness landscape of the ribosomal peptidyl transfer center (PTC) has not yet been attained. Here, we develop a method to functionally map an orthogonal tethered ribosome (oRiboT), which permits complete mutagenesis of nucleotides located in the PTC and the resulting epistatic interactions. We found that most nucleotides studied showed flexibility to mutation, and identified epistatic interactions between them, which compensate for deleterious mutations. This work provides a basis for a deeper understanding of ribosome function and malleability and could be used to inform design of engineered ribosomes with applications to synthesize next-generation biomaterials and therapeutics.

## Teaser

The fitness landscape of the ribosome is calculated by assaying translation by libraries of orthogonal tethered ribosomes.

## INTRODUCTION

All genetic elements are in a perpetual state of change via mutation and natural selection. To comprehensively understand the mechanisms of biochemical systems and evolve them for altered activity, knowledge of a specific genetic element’s potential to change and acquire new function is necessary. A fitness landscape provides such a dynamic description. Fitness landscapes are models of the sequence space of a genetic element that map how each sequence corresponds to its associated activity (*1*–*4*). This allows for an understanding of a genetic element as a dynamic entity whose sequence can be optimized via its fitness landscape to achieve desired functional activity either as a local or global maximum (*5*, *6*). Fitness landscapes have been constructed for proteins (*7*) or RNA (*8*) in isolation from their biological role in the cell, or even completely in vitro (*9*, *10*). Fitness landscapes can serve as predictive tools to guide evolution of enzymes or catalytic RNA, understand their biochemistry, and design sequences with desired characteristics. Understanding the epistatic (e.g., cooperative) interactions within a genetic element via its fitness landscape could augment an understanding of how networks of interactions between nucleobases or amino acids govern its function.

For the determination of fitness landscapes in vivo, a complex library sampling the sequence space of a gene is introduced into cells such that functional, or impaired, versions of this gene have an effect on cellular growth (*8*, *11*). Cells containing active versions of the gene outcompete those cells with an inactive or impaired version, and their frequency in the population increases (*2*, *6*). To quantify fitness landscapes, next-generation sequencing (NGS) can be used to infer the degree of enrichment in the population for each gene variant, which is proportional to its fitness. The application of these approaches has been challenging to achieve in the ribosome, since the ribosome assumes the essential role of protein synthesis in cellular function. Even point mutations are sufficient to create dominant lethal ribosome mutants, which are incapable of supporting life (*12*–*14*). This presents a formidable challenge to mapping the in vivo fitness landscape of the ribosome or its catalytic core, as the comprehensive sequence space of the ribosome cannot be sampled.

The ribosome is a molecular machine uniquely able to convert genetically encoded information into polypeptides and proteins in a template-directed manner at monomeric precision. Hence, engineering the ribosome for the synthesis of polymers incorporating abiological substrates (*15*–*22*) or to redesign the genetic code for expanded function and cellular control (*23*–*25*) has become a recent focus in the field of synthetic biology. There is extensive structural understanding of natural ribosomes (*26*–*30*). In addition, many biochemical and genetic studies have probed the ribosome, including a previous effort that mapped the essential nucleotides of the peptidyl transfer center (PTC) of natural ribosomes in *Escherichia coli* (*12*). This was accomplished by creating a degenerate library of plasmid-encoded ribosomes and introducing this library into cells to determine which mutants were tolerated and which were eliminated from the population because they were dominant lethal. This study provided information on positions in the PTC of a wild-type (WT) ribosome that cannot be modified, and those that showed flexibility to mutation. However, because ribosomes directly support cellular viability, many mutants could not be obtained within cells, preventing the sampling of the entire possible sequence space. Furthermore, this approach does not allow one to dissect the activity of the ribosome in isolation, as it must have a basal level of activity to translate the proteome of the cell. Although valuable information was gleaned from this study, a comprehensive in vivo fitness landscape for the ribosome has thus far not been determined.

An important advance in the field of ribosome biology and engineering has been the development of orthogonal tethered ribosomes (oRiboTs) (*31*, *32*). This ribosome contains an orthogonal anti–Shine-Dalgarno site (oaSD) (*23*, *33*), which allows for an orthogonal mRNA (omRNA) to initiate translation of a target protein from this ribosome and not the native host ribosome (*31*, *33*). Furthermore, the large and small subunits in this ribosome are physically tethered so that they do not exchange with native ribosomes in *E. coli*. As a result, this ribosome is insulated from translation of the host proteome and not directly responsible for maintaining cellular viability. This was demonstrated by an oRiboT ribosome bearing a dominant lethal mutation that did not affect cell viability (*31*). Together, these features allow for the direct measurement of oRiboT performance without bearing the burden of cellular viability.

In this work, we develop an evolution-selection method to study the function of oRiboT, which allowed us to construct the fitness landscape for this ribosome. This also permitted a deep mutational analysis of the PTC and exit tunnel of a ribosome. We use a set of three orthogonal proteins to empirically study the performance of libraries of oRiboT ribosomes and computationally reconstruct the fitness landscape of oRiboT from NGS data. We then validated our library predictions and were able to measure flexibility to mutation at individual PTC nucleotides and identify epistatic interactions between positions within the PTC. In addition to mapping functional interactions across the PTC, these findings provide a basis for the design of ribosome libraries based on empirical knowledge of their fitness landscape, which can aid the engineering of ribosomes with improved catalytic and polymerization capabilities (*15*, *19*, *22*, *34*, *35*).

## RESULTS

### Development and validation of a method for reconstructing the fitness landscape of the PTC and exit tunnel of oRiboT

**Fig. 1.**
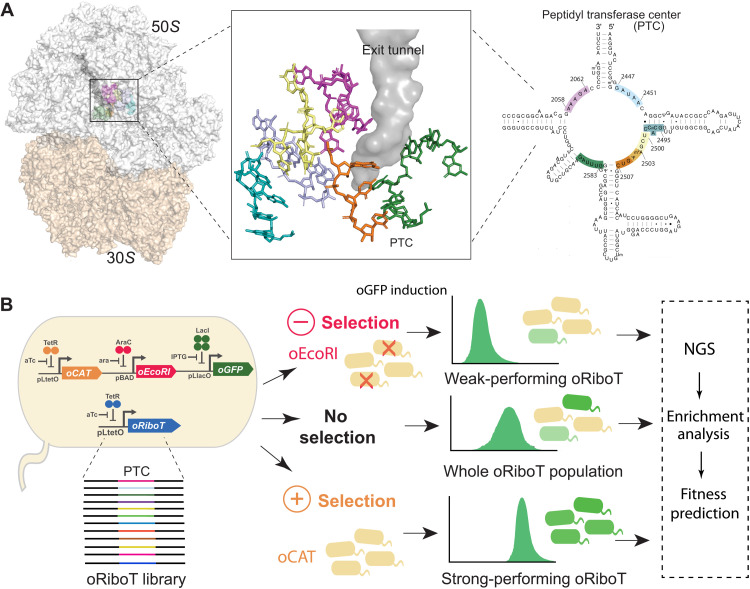
Determining the fitness landscape of orthogonal tethered ribosome (oRiboT). (**A**) Secondary structure and 3D structural model of the peptidyl transfer center (PTC)–ring nucleotides analyzed in this study. Each of the six oRiboT libraries studied is shown in a different color. The 2500 and 2503 libraries contain an intersection shown in light orange. (**B**) Workflow for the empirical/computational determination of the oRiboT fitness landscape. An oRiboT plasmid library is introduced into a strain having a plasmid containing orthogonal EcoRI (oEcoRI) (red), orthogonal CAT (oCAT) (orange), and orthogonal GFP (oGFP) (green). This population is then subjected to negative selection with oEcoRI and positive selection with oCAT. Weak-performing ribosomes are selected for in the oEcoRI negative selection, and strong-performing ribosomes are enriched in the positive oCAT selection. oRiboT plasmids are then isolated from the positive selection, negative selection, and unselected populations. Upon sequencing with next-generation sequencing (NGS), the enrichments of mutants in positive and negative selections as compared to the original populations are computed and used to reconstruct the fitness of the ribosomes. “o” denotes orthogonal mRNA.

We chose to study the PTC and exit tunnel entrance of the ribosome, as this is the most conserved portion of the ribosome and fundamental for catalysis of peptide bond formation. For this reason, it is also least permissive for mutagenesis in native ribosomes, due to the generation of dominant lethal mutations (*12*). It is also the focus of ongoing research to engineer ribosomes to catalyze the polymerization of abiological chemistries, and thus, an understanding of the fitness landscape of oRiboT would be valuable for these goals. We chose to study nucleotides 15 Å away from the A/P site, as these nucleotides are highly conserved and believed to be essential for peptide bond formation. We divided the PTC ring into six libraries of five or six nucleotides each to obtain full library depth upon NGS (Fig. 1A).

To determine the fitness landscape of oRiboT, we developed a strategy that relies on the output of three orthogonal genes contained on a single plasmid in *E. coli*. We constructed an oEcoRI-oCAT-oGFP selection/screen plasmid where each gene, EcoRI, GFP, and CAT is regulated by its own inducible promoter (fig. S1). We drove the expression of orthogonal EcoRI (oEcoRI) from an arabinose-inducible promoter (pBAD) and orthogonal CAT (oCAT) from an anhydrotetracycline (aTc)–inducible promoter (PL-tetO). We controlled the expression of orthogonal GFP (oGFP) from the isopropyl-β-d-thiogalactopyranoside (IPTG)–LacI-inducible PL-lacO promoter. Expression of oRiboT on its own plasmid was controlled from the aTc–TetR-inducible PL-tetO promoter (fig. S1). EcoRI, a toxic protein (*36*), causes cell death when its gene (oEcoRI) is translated by oRiboT. However, if the oRiboT mutant is impaired or nonfunctional, it will attenuate production of EcoRI. Thus, there is a selection for impaired or nonfunctional oRiboT mutants, and we hypothesized that these would be enriched in the population after oEcoRI induction (Fig. 1B). Similarly, in the oCAT-positive selection, oRiboT needs to translate the CAT enzyme that catalyzes chloramphenicol acetylation to survive and replicate in medium containing chloramphenicol. Nonfunctional or impaired oRiboT mutants will die in the chloramphenicol selection, while oRiboT mutants that are capable of effectively translating oCAT will be enriched (Fig. 1B). This creates a selection for active oRiboT mutants. Similarly, functional oRiboT mutants will be able to translate oGFP, while nonfunctional or impaired oRiboT mutants would be expected to translate lower levels of oGFP. We thus hypothesized that negative selection by oEcoRI should be inversely correlated with the ability to translate oGFP, and positive selection by oCAT would be positively correlated with the ability to translate oGFP. Thus, the last step in our approach entails inducing oGFP in the selected populations and performing a fluorescence-activated cell sorting (FACS) sort for nonfluorescent cells after the oEcoRI selection and FACS sort for fluorescent cells after the oCAT selection (Fig. 1B). At the conclusion of these sorts, the oRiboT library composition within both the oGFP-positive and oGFP-negative populations is the sum of the translation of two separate orthogonal proteins in each condition. Thus, in total, oRiboT function is assayed by the translation of three orthogonal proteins. This ensures that oRiboT activity is not biased by the translation of a single protein. Subsequently, the negative and positive sorted populations, as well as the original oRiboT library, are sequenced with NGS, and the enrichments of each oRiboT mutant in the library are quantified (Fig. 1B). The ratio between the degree of enrichment of each oRiboT mutant in positive to negative selections, compared to the unselected populations, is used to predict the fitness of each oRiboT mutant. This approach is similar to previous methods where the degree of enrichment of members of a complex library of a genetic element subjected to selection was used to reconstruct its fitness landscape (*2*, *6*, *8*, *11*). The use of parallel positive and negative selections we develop in this study is uniquely enabled by oRiboT, as we hypothesized that we could obtain not only enrichment of functional oRiboT variants in positive selections but also enrichment of nonfunctional oRiboT variants in negative selections, thereby expanding the resolution of our fitness landscape determination.

To initially test the operation of our positive and negative selections, we constructed an oRiboT plasmid library with full randomization of nucleotides 2058–2062 (exit tunnel entrance) using QuikChange lightning mutagenesis and introduced this library into *E. coli* cells containing the oEcoRI-oCAT-oGFP selection/screen plasmid (fig. S1). We characterized the library by NGS (figs. S2 and S3) and found that we achieved full theoretical complexity (i.e., 1024) and did not observe biases of nucleobases at each position in the library (figs. S2A and S3A). We measured kinetic growth curves of cells containing the 2058–2062 oRiboT library and triple orthogonal gene plasmid under full induction of oEcoRI (+arabinose) and oRiboT (+aTc). We hypothesized that we would observe an enrichment of nonfunctional oRiboT mutants and improved growth of the degenerate ribosome population relative to WT. We tested two concentrations of aTc (100 and 400 ng/ml) to determine the optimal conditions for use in this selection. We observed reduced toxicity compared to growth with WT oRiboT and oEcoRI induction in both conditions (fig. S4A), suggesting that impaired oRiboT mutants were able to escape oEcoRI selection and accumulate in the population. To assay the activity of the oRiboT mutants in the population, we induced oRiboT (+aTc) and oGFP (+IPTG) expression. The level of oGFP production can be a metric of oRiboT function (*31*, *37*). We observed that in comparison to the original 2058–2062 library, which had a broad distribution of oGFP fluorescence compared to WT oRiboT, the population after oEcoRI selection was mostly nonfluorescent (fig. S4B). This demonstrates that nonfunctional oRiboT mutants were selected in this negative selection. Furthermore, we sequenced this population with NGS and determined that 100% of the 1024 original library members were still present. This was encouraging, as this validated that we could shift the frequency of the population with this negative selection but still retain all members of the population, a criterion that we hypothesized would allow us to reconstruct the full fitness landscape of this library (*9*, *38*). In comparing the two concentrations of aTc (100 and 400 ng/ml) we used to determine the optimal selection conditions above, we observed a qualitatively similar outcome in the 400 ng/ml aTc condition as in the 100 ng/ml aTc condition. We thus chose to use the 100 ng/ml aTc concentration for further experiments.

To determine the optimal conditions for oCAT to enrich for functional oRiboT mutants in a positive selection, we grew the strain containing the 2058–2062 oRiboT library and oEcoRI-oCAT-oGFP selection/screen plasmid under full induction of oRiboT and oCAT (+aTc), along with 0, 62, 124, and 248 μM chloramphenicol, and measured kinetic growth curves (fig. S5). Since functional oCAT is necessary to rescue the cell from chloramphenicol toxicity, we also varied the induction time of oCAT between 0.5, 1, and 2 hours before chloramphenicol addition to determine the optimal time to accumulate the CAT enzyme. We measured the growth curves of these populations after addition of 0, 62, 124, and 248 μM chloramphenicol. We then induced oGFP (+IPTG) and oRiboT (+aTc) in the populations that survived to assess the oRiboT function of library members, as before. We observed that there was improved growth with 2-hour preinduction and 248 μM chloramphenicol compared to the other conditions (fig. S5, D to F). Also, the 2-hour, 248 μM chloramphenicol, condition demonstrated a unimodal oGFP-positive population (fig. S5C), in contrast to the other conditions (fig. S5, A and B). The 0.5- and 1-hour time points showed notably delayed growth compared to the 2-hour preinduction (fig. S5, D to F). Subsequent sequencing with NGS on the 2-hour preinduced, 248 μM chloramphenicol, population confirmed that all mutants of the library were captured after selection. This indicates that the selection in this condition was not overly stringent, resulting in no loss of population members. This is desired, since we would want full population coverage to quantify relative rates of enrichment of each oRiboT mutant and the averaged contribution of each PTC nucleotide to this enrichment, which is not possible when oRiboT mutants are absent from the population. The oGFP data for this population (fig. S5C) illustrate that the population was enriched for functional oRiboT mutants after initially being a mixed population of functional and nonfunctional oRiboT mutants. On the basis of the performance of the 2-hour preinduction and 248 μM chloramphenicol growth condition, we chose to proceed with this set of conditions for further experiments.

### Determination of the fitness of oRiboT libraries spanning the PTC and exit tunnel

To determine the fitness of the oRiboT mutants in the 2058–2062 library, we performed positive and negative selections with our optimized selection conditions and then sorted the negative oEcoRI selection for negative oGFP fluorescence, and positive oCAT selection for positive oGFP fluorescence using FACS. We then purified the plasmids containing libraries of oRiboT ribosomes from each condition and performed sequencing by NGS, as well as on the original population that had not undergone selections. We obtained the level of enrichment of each oRiboT mutant in the population for positive and negative selections by subtracting number of reads of each ribosome sequence in the selection condition from the corresponding sequence in the unselected population (fig. S6, Materials and Methods) (*7*, *39*). We computed the fitness of each oRiboT mutant as a ratio of positive to negative selection enrichment (Materials and Methods). We found that the fitness computed for all oRiboT mutants in the population formed a normal distribution (fig. S7A). To determine the degree to which each nucleotide substitution at each position from 2058–2062 in the exit tunnel entrance contributes to oRiboT fitness, we produced a heatmap of enrichment for each of the four nucleotides at each position (fig. S8A). We observed that at position A2060, adenosine (A) was strongly enriched and all other mutations were disfavored. This supports previous literature on A2060 being a dominant lethal position (*12*, *40*). G2061 (also a dominant lethal position) (*12*) was also strongly preferred, but we also saw enrichment for cytidine (C) (fig. S8A). Previous analysis of this mutant in *E. coli* ribosomes demonstrated that it not only catalyzes peptide bond formation in vitro but also has an effect on the *E. coli* stress response (*41*). This may explain the negative effect on cellular viability if this mutation is found in natural ribosomes but not oRiboT. Analysis of the other bases in this library showed A2058G, A2059G, and A2062U mutations being enriched. All three mutants have previously been previously reported to be functional and able to confer antibiotic resistance in WT ribosomes (*42*, *43*).

**Fig. 2.**
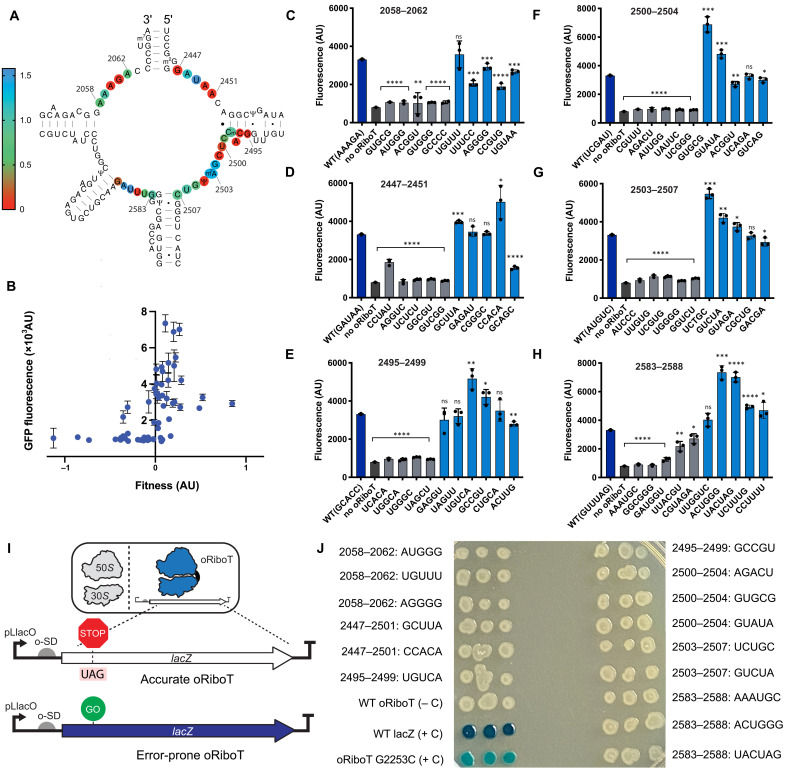
The fitness landscape of orthogonal tethered ribosome (oRiboT) and validation of mutation across all libraries studied. (**A**) We determined the flexibility toward mutation at eachpeptidyl transfer center (PTC) base by computing the Shannon entropy at each site given enrichment of each base at that position. The entropy at each position is plotted in bits from 0 to 2, with 2 being maximum flexibility. (**B** to **H**) After fluorescence-activated cell sorting (FACS) sorting each of the six libraries for low and high orthogonal GFP (oGFP)–producing mutants, we analyzed endpoint fluorescence and performed Sanger sequencing of each clone. (B) We compared the predicted fitness of each mutant to its oGFP fluorescence. (C to H) Endpoint fluorescence for all clones obtained from the FACS experiment and plotted for the (C) 2058–2062 library, (D) 2447–2451 library, (E) 2495–2499 library, (F) 2500–2504 library, (G) 2503–2507 library, and (H) 2583–2588 library. Gray bars indicate clones from the negative FACS sort, and blue bars indicate clones from the positive FACS sort. Data represent *n* = 3 independent experiments, with SD shown. A Student’s *t* test was used to calculate statistical significance relative to wild-type (WT) oRiboT (dark blue), where ns (not significant) = *P* > 0.05, *P < 0.05, **P < 0.01, ***P < 0.001, and ****P < 0.0001. (**I**) We used an orthogonal lacZ (o-lacZ) assay to measure the global fidelity of translation of the oRiboT mutants from (C) to (H). Readthrough of lacZ containing a premature stop codon (UAG) by error-prone oRiboT should produce a blue plaque, whereas accurate oRiboT translation should produce a white plaque. (**J**) We retransformed the select oRiboT mutants profiled in (C) to (H) into *E. coli* cells containing the orthogonal o-lacZ plasmid and plated them or control clones on plates containing 5-bromo-4-chloro-3-indolyl-β-d-galactopyranoside (X-Gal), isopropyl-β-D-thiogalactopyranoside (IPTG), and anhydrotetracycline (aTc). A photograph of the agar plate, containing biological replicates (*n* = 3) for each condition, is shown.

We next computed the Shannon entropy (*44*) at each position (Materials and Methods) to quantify the flexibility of mutations at each site (*10*, *45*). We found positions A2058, A2059, and G2061 to be permissive to mutations beyond their WT base (Shannon entropy > 0), while positions A2060 and A2062 have an entropy of 0, indicative of little flexibility to mutation (fig. S8G). We mapped the entropy values as a heatmap to positions A2058–A2062 of the PTC (Fig. 2A). Positions A2060 and A2062 are highly inflexible to mutation (red), and the remaining positions are moderately flexible (green), indicating that they support a limited repertoire of mutations while still maintaining ribosome function. Previous work on natural ribosomes identified that positions A2060 and G2061 are dominant lethal mutations, leading to nonfunctional ribosomes. We observed that while our predictions showed those sites to have reduced activity upon mutation compared to the other positions in this library, there were mutants that were enriched in the positive selection and depleted in the negative selection having mutations in those positions (fig. S8A). These data suggest that mutations at other sites can help alleviate deleterious oRiboT function through compensation, even at highly inflexible positions.

We constructed six additional libraries with QuikChange Lightning mutagenesis having full randomization of nucleotides across five regions (2447–2451, 2495–2499, 2500–2504, 2503–2507, or 2583–2588) and introduced them into cells containing the oEcoRI-oCAT-oGFP selection/screen plasmid. Using the optimized selection conditions described above (figs. S2, S3, S7, and S8), we performed positive and negative selections and sorted the negative oEcoRI selection for negative oGFP fluorescence, and positive oCAT selection for positive oGFP fluorescence using FACS. Next, we performed NGS on the purified plasmids containing libraries of oRiboT ribosomes from each selection condition, as well as on the original population that had not undergone selections. Enrichment of nucleobases at positions throughout the PTC (fig. S8) matched many findings from previous reports (*12*, *42*, *43*, *46*–*49*). For example, we found that G2583C was strongly selected against (fig. S8F). It is known that this mutant leads to decreased total protein synthesis and an increase in peptidyl–transfer RNA (tRNA) drop-off, reducing translational processivity (*50*, *51*). In addition, we provide data on PTC mutations that were not previously characterized in vivo, such as mutations in bases C2501, G2502, C2507, and C2587 (Fig. 2 and fig. S8). C2501 forms a conserved wobble pair with A2450 and is implicated in maintaining the tertiary structure near the catalytic center (*52*), while C2507 has a potential role in stabilizing the positioning of the tRNA 3′ end in the A site (*12*).

Upon mapping the entropy of all the studied bases as a heatmap to the PTC, we observed that there are bases exhibiting high mutational flexibility (blue), intermediate mutational flexibility (green), and low mutational flexibility (red) (Fig. 2A). Most bases profiled in this study displayed intermediate mutational flexibility (green), wherein they contain only a limited repertoire of mutations by which they maintained ribosome activity. A minority of bases were determined to be highly flexible (blue) or highly inflexible to mutation (red), respectively (Fig. 2A and table S1). Most of the highly inflexible bases matched previously reported dominant lethal mutants (i.e., A2060, A2450, G2495, U2500, and C2507) (*12*, *53*).

Some previously reported dominant negative mutants (*12*) showed medium-to-high flexibility to mutation (e.g., A2451, U2504, G2505, and U2586). In a previous study (*46*), ribosomes containing these mutations exhibited flexibility to mutation without loss of function. Notably, in a previous characterization of oRiboT mutants harboring A2451C and A2451U mutations (*31*), these ribosomes exhibited robust protein synthesis in vivo. This matches our findings that U and C are highly enriched for oRiboT fitness at position 2451, although A2451C and A2451U are known dominant lethal mutants (fig. S8B). These findings are particularly important given that the oRiboT activity predictions from our method match previously reported characterization of the oRiboT ribosome. In addition, our results and previous work (*31*) on oRiboT highlight that unique sequence space can be accessed in oRiboT that is inaccessible to natural ribosomes, and these ribosomal mutants may have unique and robust functionality. This may partially be explained by the ability to insulate oRiboT from the cell’s native translation system, which has many regulatory interactions in the cell (*41*, *54*, *55*) besides supporting the cell’s proteome (e.g., regulation of stringent response or Sec protein translocation pathway). These findings highlight the significance of our efforts to map the fitness landscape of the oRiboT ribosome.

To determine the degree of reproducibility between independent selections performed on the same library, we performed selections for libraries 2058–2062, 2447–2501, and 2495–2499 in duplicate and performed sequencing with NGS on these libraries. We computed the enrichment of all four nucleotides at each site in each library. We found that the patterns of enrichment were significant and highly reproducible between independent selections (fig. S9, A to C). Furthermore, we sought to determine the degree of reproducibility of oRiboT fitness predictions when two independently constructed libraries, using separate cloning methodologies, were subjected to our optimized selections and sorts. We constructed the 2583–2588 library using QuikChange Lightning or Gibson assembly (Materials and Methods). The two naïve libraries contained notable differences in their frequency of nucleotides at each position from 2583–2588 (fig. S10A). However, after we performed the same selection method as before, we found high reproducibility between the nucleotides enriched at each site of the library (*R*^2^ = 0.8961, *P* < 0.0001) (fig. S10B). This suggests that our method is capable of reconstructing the same fitness landscape even when the ratios of oRiboT mutants in the nonselected starting libraries differ substantially.

### Validation of fitness predictions in each library

To validate the fitness predictions from our analyses of PTC libraries, we conducted an experiment to study predicted fitness and compared it to oGFP production by oRiboT. We grew each of the six libraries with induction of oRiboT (+aTc) and oGFP (+IPTG) and sorted each of the six libraries into positive and negative bins with FACS. We then obtained five unique single clones from each positive and negative bin. We calculated the fitness of each sequence as before and grew each clone in triplicate for 16 hours to obtain endpoint oGFP fluorescence (Fig. 2, C to H). We found that the sequences that had high oGFP expression had high fitness predictions and most low-fluorescence clones had negative fitness predictions (Fig. 2B). These results support the fitness values and flexibility to mutations and provide predictive insight into oRiboT activity of mutations in oRiboT PTC sites (Fig. 2A).

Since we observed high oRiboT activity in mutants harboring alternations to the most conserved bases within the PTC and exit tunnel entrance, we sought to further investigate whether the accuracy of translation was affected. We constructed an orthogonal lacZ (o-lacZ) reporter plasmid containing a premature UAG stop codon previously reported to discriminate error-prone translation (*56*) and used it for blue/white colorimetric detection of oRiboT fidelity (Fig. 2I). A β-galactosidase ribosome fidelity assay has been used in numerous studies for measurement of accuracy of translation (*56*–*60*). The lacZ gene coding for β-galactosidase catalyzes the conversion of 5-bromo-4-chloro-3-indolyl-β-d-galactopyranoside (X-Gal) into a blue dye that can be detected as a colorimetric readout (*31*, *56*–*60*). We constructed a plasmid with o-lacZ containing a premature stop codon. In the case of accurate ribosome translation of o-lacZ, a truncated β-galactosidase protein will be translated, and no blue pigment will be produced. However, in the case of translation of o-lacZ by error-prone ribosomes, the premature stop codon will be skipped through frameshifting or amino acid misincorporation, and a full-length β-galactosidase enzyme will be produced, which will convert X-Gal into a blue dye. We picked two mutants from each oRiboT library we profiled for oGFP expression that showed robust oGFP translation and three additional plasmids that had low oGFP translation (Fig. 2, C to H) and retransformed these 15 plasmids into *E. coli* cells containing the o-lacZ plasmid. We then plated the cells on plates containing X-Gal, and inducers for o-lacZ (IPTG) and oRiboT (aTc), and also plated a WT oRiboT + o-lacZ plasmid negative control, a WT o-lacZ plasmid (lacking premature UAG stop codon) + oRiboT positive control, as well as an oRiboT G2253C mutant + o-lacZ plasmid positive control. The G2253C mutation has been shown in WT ribosomes to reduce translational accuracy and lead to increased readthrough of stop codons (*61*), which is a positive control for the error-prone translation of o-LacZ. We observed that the WT o-lacZ plasmid positive control generated robust blue plaques. The G2253C oRiboT positive control (with o-lacZ plasmid) also generated blue plaques that were fainter in color (Fig. 2J), which would be expected as stop codon readthrough would not be as efficient as the translation of a full-length protein without a premature stop codon (i.e., WT o-lacZ). All other conditions contained white plaques (Fig. 2J). These results indicate that no detectable levels of stop codon readthrough were detected in the oRiboT mutants profiled. The oRiboT mutants that produced no detectable oGFP could possibly be error-prone, but they would also not be detectable with this assay due to being inactive in protein translation. These data suggest that we are able to identify highly active or inactive oRiboT mutants, and the activity of these mutants matches our fitness landscape predictions (Fig. 2B).

### Determination of pairwise fitness relationships of bases comprising the PTC and exit tunnel

Encouraged by the ability to map the fitness effects of individual bases throughout the catalytic core of oRiboT, we proceeded to determine the pairwise fitness relationships (*8*) of bases comprising the PTC and exit tunnel entrance. We first examined all possible combinations of two positions in each library and their effect on the fitness of oRiboT and created heatmaps of the enrichment at each pair of nucleobase combinations (fig. S11). This analysis of oRiboT function importantly expands on the previous functional models of the ribosome, which purely examined the contribution or essentiality of each individual PTC base to ribosome function (*12*, *46*). Notably, as seen in the 2058–2062 library, the nucleotide G2061 is generally resistant to mutation. The G2061C mutation is known to be dominant lethal (*12*), and our analysis confirms that, generally, a mutation to C at this position results in nonfunctional ribosomes. However, when there is also a G at position A2058 or A2059, we observe improved oRiboT fitness with C at position 2061 (fig. S11A). We see analogous results at base U2504, which is highly conserved among all domains of life (*46*). The U2504G mutation is dominantly lethal (*12*), and in our analysis, we observe a strong preference for uridine (U) at this position (fig. S6E). However, when examined with covariation with other sites, we observe that G is enriched at base U2504 if there is an A at base C2501, or an A or U at base G2502, or a U or G at base A2503 (fig. S11D). These results highlight how the flexibility to mutation that is observed in the ribosome can also be explained by compensation via epistasis with neighboring nucleotides. We observe several instances of plasticity at the most conserved bases in the PTC of oRiboT (Fig. 2A and fig. S11).

**Fig. 3.**
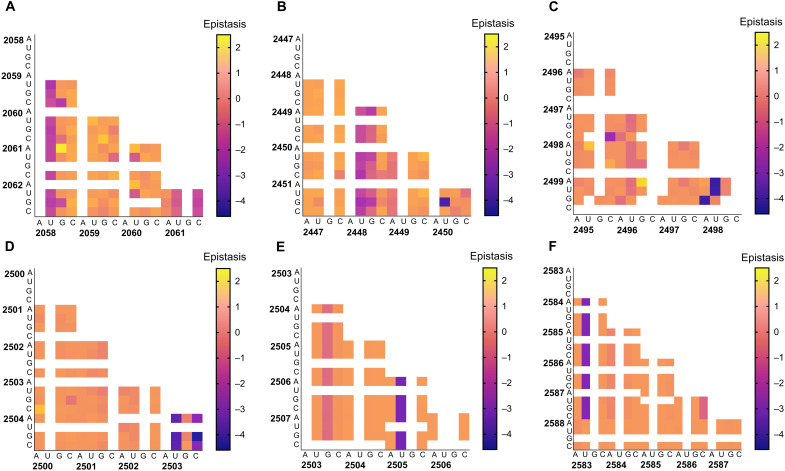
Epistasis between bases across the peptidyl transfer center (PTC). The epistasis between all dual mutants was examined across each of the orthogonal tethered ribosome (oRiboT) libraries to measure the change in fitness between a pair of any possible ribosome point mutants, to the fitness of the resultant dual mutant composed of these two point mutations. The heatmaps of epistasis between the sites of each library were plotted for the (**A**) 2058–2062 library, (**B**) 2447–2451 library, (**C**) 2495–2499 library, (**D**) 2500–2504 library, (**E**) 2503–2507 library, and (**F**) 2583–2588 library. Negative epistasis indicates a negative fitness upon mutation of the two nucleotide positions, an epistasis of 0 indicates a neutral fitness effect upon mutation, and a positive epistasis indicates a positive fitness upon mutation of the two positions.

We then expanded the above analysis to study epistatic interactions between all pairwise positions in each library. We computed the fitness values of all possible dual mutants and subtracted from them the product of their constituent point mutants’ fitness values (Materials and Methods). This measure of epistasis examines the change in fitness between a pair of any possible ribosome point mutants to the fitness of the resultant dual mutant composed of these two-point mutations. From these data, we generated heatmaps of pairwise epistasis for all bases at each position in each library (Fig. 3, A to F). We found that mutations at bases G2061 and A2062 are negatively correlated, especially upon introduction of U or C at each base (Fig. 3A). As nucleotide 2061 is a dominant lethal site (*12*), it could be inflexible to mutation and influence its neighboring base. We found that nucleotide 2061 had intermediate flexibility and nucleotide 2062 was highly inflexible (Fig. 2A), which further supports a possible functional role. Previous molecular dynamics modeling proposes an allosteric relationship between these nucleotides (*62*). We also observed how nucleotide A2448 has a positive epistatic relationship with nucleotides 2449 to 2451 when mutated to A2448C, but otherwise maintains a negative epistasis with these nucleotides (Fig. 3B). There is also a strong negative epistasis between nucleotides A2503 and U2504 when A2503 is mutated to U or C (Fig. 3D). These data compliment the flexible sites of mutation in oRiboT we previously observed (fig. S11). For example, we observed how G is enriched at nucleotide U2504 if there is an A or U at position 2502, or a U or G at position 2503. An epistatic relationship between these nucleotides would explain why variation in one or the other would necessitate a compensatory change in another specific nucleotide. In previous work, the A2503U and U2504G mutations separately conferred resistance to multiple macrolide antibiotics (*63*). Collectively, these results provide a view of the PTC that examines individual nucleotides and their essentiality and also provides hotspots of interaction between nucleotides across the PTC. This allows for prediction of which sequences could compensate for nonfunctional mutations at previously dominant lethal sites. However, we should note that additional hydrogen bonding and Watson-Crick interactions exist with bases not explored simultaneously in this study. For example, both A2503 and U2504 interact with base A2058 (*63*), which was separately explored in the A2058–2062 library. Future exploration of key positions between the sites we studied could yield even more detailed picture of epistatic interactions across the PTC in future studies.

### Three-dimensional modeling of the ribosome’s PTC and exit tunnel entrance to understand mutational flexibility and epistasis

We next sought to map our analysis of the mutational flexibility and epistasis within oRiboT onto the ribosome’s three-dimensional (3D) structure to generate a functional map of the ribosome’s active site. We began by examining how distance of PTC nucleotides from the elongating peptide beginning in the PTC and continuing to the exit tunnel affects their mutational flexibility. We used a cryo–electron microscopy structure of the SecM peptide stalled in the ribosomal exit tunnel [Protein Data Bank (PDB) ID: 3JBV] (*64*) as a model for a general elongating peptide in the ribosome during translation.

**Fig. 4.**
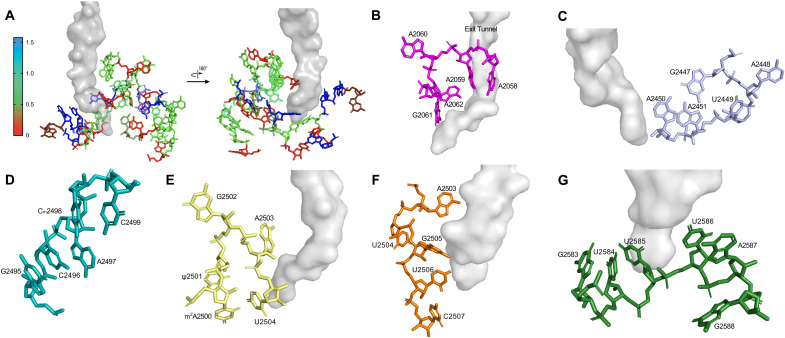
Structural modeling of the ribosome’s peptidyl transfer center (PTC) and interactions with elongating peptide support fitness and epistatic predictions. (**A**) Structural model of all of the ribosome nucleotides studied represented as a heatmap of flexibility to mutation (Shannon entropy) next to elongating peptide (gray surface). (**B**) Structural model of the 2058–2062 exit tunnel library. (**C** to **G**) Structural models of the PTC libraries including (C) bases 2447–2451, (D) bases 2495–2499, (E) bases 2500–2504, (F) bases 2503–2507 (G), and bases 2583–2588. Ribosome structure accessed from PDB ID: 3JBV. The colors in (B) to (G) correspond to the color codes of the libraries studied, as in Fig. 1A.

We previously determined that the PTC and exit tunnel entrance have regions of high mutational flexibility (blue), medium mutational flexibility (green), and low mutational flexibility (red) (Fig. 2A). We mapped these as a 3D heatmap (Fig. 4A) and found that there was excellent structural agreement with our predictions. Most of the nucleotides that had low flexibility to mutation were in close proximity to the elongating peptide (e.g., A2060, A2062, A2450, C2507, and U2584) (Fig. 4A). Also, many of the nucleotides with intermediate flexibility (green) form a pocket between the highly inflexible bases (red) in close proximity to the elongating peptide and the highly flexible nucleotides (blue), which are either further from the peptide or in the front wall of the PTC (Fig. 4A).

However, in certain positions (e.g., A2451), there was intermediate flexibility to mutation even in close proximity to the elongating peptide, favoring introduction of pyrimidine (C or U) instead of purine (A), which would allow more space, and hence flexibility to the peptide in motion through the exit tunnel (Fig. 4C). As previously noted, prior work on the oRiboT ribosome had also shown that the A2451C and A2451U ribosome mutants were highly active in vivo (*31*). The 2451 and 2452 bases are located within the PTC in immediate proximity to the start of the peptide (Fig. 4C) and are known to be next to the tRNA-P site, being essential in the peptidyl-transferase reaction (*65*). This purine to pyrimidine swap could also provide more space in the A/P site for peptide bond formation. In addition, the epistatic predictions for nucleotides 2447–2451 match structural modeling, as A2450U and A2451G show negative epistasis but the same fitness decrease is not observed for pyrimidine substitution at A2451 (A2451C or A2451U with A2450), which further supports the hypothesized fitness benefit of having more flexibility in this PTC position (Fig. 4C).

Position 2062 in the exit tunnel entrance is highly inflexible to mutation (Figs. 2A and 4A), but a U is favored over the WT A (fig. S8A). We also observed enrichment for A2058G and A2059G mutations in conferring improved oRiboT fitness (fig. S8A). The A2058G, A2059G, and A2062U mutants have been demonstrated previously to confer macrolide resistance in *E. coli* (*42*, *43*). In addition, A2058 methylation has been shown to confer macrolide resistance (*66*), which would have an analogous structural effect to a G substitution at that position. All three bases are located at the narrowest point of the exit tunnel (*67*) (Fig. 4B and fig. S12). These data suggest that there is significance to altering this point in the exit tunnel to improve oRiboT fitness. In particular, the A2062U purine to pyrimidine conversion would lead to widening of this narrow point in the exit tunnel.

The examination of the structural mapping of our fitness landscape of oRiboT allowed us to develop an understanding of the mutational flexibility and epistasis between nucleotides within oRiboT grounded in ribosome structure. In nucleotides 2495–2499, we observed that 2496 and 2497, as well as 2496 and 2498, had negative epistasis with each other, which would be reasonable considering their proximity (Fig. 4D). Similarly, a negative epistatic relationship is observed between nucleotides 2503 and 2504. Bases 2501 and 2503, and 2501 and 2504 exhibited not as pronounced an epistasis (Figs. 3D and 4E). We observed a fitness gain over background when A2503G and U2504A, A2503 and U2504G, or A2503G and U2504G mutations coexist (fig. S11D). These mutations would introduce narrowing of the bases around the elongating peptide in this position (Fig. 4E). This would suggest that narrowing here improves translation by oRiboT via tighter control over translation. The important role of these two bases is highlighted by the fitness cost upon mutating either of them (i.e., strong negative epistasis) (Fig. 3D). It is known that A2503 plays a role in antibiotic response through nascent peptide stalling of several peptide sequences responsible for antibiotic resistance, and the A2503G mutation leads to reduced translational stalling of a number of these peptides (*68*). This would suggest that mutation at this base could reduce ribosomal specificity for the peptide sequence translated. Similarly, base U2504 shows very species-specific response to antibiotics; the U2504G mutation has been shown to confer resistance to several antibiotics (*63*). This suggests that cells harboring this ribosomal mutation may exhibit a fitness improvement. Together, these data suggest a potential benefit to ribosomal activity in not maintaining such a stringent control over translation at this site, especially when it is no longer used for antibiotic-specific defense mechanism in nature. These findings, when examined along with the fitness benefit of widening at bases 2451 and 2062, indicate that there are potential regions in the ribosome’s catalytic core that benefit from tighter control and others that benefit from increased flexibility.

Within the 2503–2507 library, we observed that nucleotides 2504, 2505, 2506, and 2507 show strong preference for their WT bases (fig. S8E) due to their proximity to the translating peptide (Fig. 4F). In A2503, which forms a strong interaction with the peptide (Fig. 4F), the WT A is strongly preferred (fig. S8, D and E), although this is not a dominant lethal mutation site in *E. coli* (*12*, *69*). Within the 2583–2588 library, both bases U2585 and U2586 are known dominant lethal sites (*12*, *70*). In U2586, despite its flexibility to mutation, G is strongly disfavored, and in U2585 (intermediate flexibility), A and G are both disfavored, indicating that narrowing of the space here would be detrimental to ribosome function (Fig. 4G). In positions 2585 to 2588, G was disfavored (Fig. 4G), suggesting a preference for higher flexibility in this location. Furthermore, nucleotide U2585 showed nonzero epistatic interactions with other bases, regardless of the base substitution at 2585 (Fig. 3F), which supports its important role in translation, as previously reported (*71*, *72*). It is known that U2585 is a universally conserved base and interacts with the CCA acceptor stem of tRNA in the P site (*13*, *73*).

## DISCUSSION

In this study, we have developed a method to mutagenize the catalytic center of oRiboT ribosomes and computationally map the sequences of individual oRiboT mutants to their fitness. Using this method, we determined the fitness landscape within the PTC and exit tunnel entrance of oRiboT, including the epistatic relationships between bases in those regions. Unlike previous approaches to study the essentiality of nucleotides in the PTC of natural ribosomes (*12*), we were able to sample all possible mutations, which allowed us to create a comprehensive fitness landscape for oRiboT. Our approach builds off of prior efforts for fitness landscape reconstruction (*2*, *6*, *8*, *11*). In these studies, active variants of a gene increase in frequency as a result of selection, and this is used to determine the degree of enrichment in the population for each gene variant using NGS, which is proportional to its fitness. However, because we used an orthogonal translation system, we have the ability to enrich for both less active and more active ribosomes through separate positive and negative selections on a population of randomized oRiboT mutants. Together, this information enabled more accurate determination of the fitness landscape of oRiboT, as we could examine the extent to which each oRiboT mutant was enriched in positive selections and depleted in negative selections.

The evolution-selection method used in this study allowed us to generate a comprehensive mutational landscape of the ribosome with the ability to understand how fitness can be influenced by epistatic interactions between pairs of nucleotides throughout the PTC. Through the generation of combinatorial mutations across the PTC, we were able to examine mutations individually as in previous work (*12*, *46*) and at higher-order combinations to study how multiple sites within the PTC map to oRiboT function. This analysis allowed us to explore the malleability of the ribosome. We found that some of the most conserved ribosomal nucleotides, which were previously shown to be inflexible to mutation, can be mutated to yield functional tethered ribosomes, provided that specified nucleotides sharing positive epistasis are simultaneously mutated (Figs. 2A and 4A). However, despite this flexibility to mutation, we also note that there are specific sites within the PTC that are highly inflexible to mutation, such as bases 2060, 2062, 2450, and 2507. This information is particularly valuable for designing more efficient oRiboT libraries for ribosome evolution efforts.

While the fitness landscape illuminates important epistatic interactions between PTC bases, we should note that structural differences between natural ribosomes and oRiboT could cause subtle differences in ribosome function (*31*, *37*, *74*). Thus, this work should be interpreted within the context of oRiboT. This could underlie why certain mutations (e.g., A2451C) were previously shown to be flexible to mutation in oRiboT (*31*) and also predicted to be functional in our study, but were dominant lethal in natural ribosomes (*75*). In addition, while this work determined epistatic interactions between positions throughout the PTC, we should note that Watson-Crick pairs and hydrogen bonding interactions exist between bases we studied and those excluded from this study. Future studies could explore epistasis between the regions studied in this work to create an even more robust model of ribosome function. Also, another limitation is that we are examining production of oEcoRI, oCAT, and oGFP proteins and not the entire proteome. Thus, ribosome fitness is the general “activity” of the ribosome, and provides a global picture, but removed from the context of natural ribosomes in their biological role. However, this is also one of the advantages of this study, as we demonstrate the ability to mutagenize nucleotides in the oRiboT ribosome that directly affect translation, separated from the constraints of WT ribosomes. In doing so, this fitness landscape permits additional information that reveals specific nucleotides and combinations of nucleotides that most directly affect translation of proteins. These insights are not attainable with analogous studies of WT ribosomes. Further improvements on this methodology could integrate mechanistic examination of oRiboT mutants into the workflow. However, information gathered from a fitness of oRiboT alone could be suitable for production of proteins or protein biopolymers that are independent of natural proteomics and unrelated to essential cellular functions.

By constructing the fitness landscape of oRiboT, this work enhances our understanding of one of the most complex macromolecular machines in nature. The approach developed in this work could set the stage both for studies to more comprehensively understand ribosome function and applications to reengineer the ribosome for expanded function. The ability to predict functional ribosome variants from fitness maps could minimize nonfunctional variants and generate ribosome libraries that probe the deepest possible sequence space, allowing for more rational evolution efforts. This work could inform the evolution of ribosomes with expanded catalytic capabilities for the biosynthesis of abiological polymers (*15*, *19*, *22*, *34*, *35*), specialized ribosomes for metabolic encapsulation (*24*, *76*), and synthesis of previously undeveloped materials and therapeutics.

## MATERIALS AND METHODS

### Strains and culture conditions

The EcNR1 strain {(*ybhB*-*bioAB*):[*cI857*Δ(*cro-ea59*):*tetR-bla*]}, modified from *E. coli K-12 substr. MG1655* as previously described (*77*), was used for experiments in this study. EcNR1 was grown in low-salt LB-min medium (10 g of tryptone, 5 g of yeast extract, and 5 g of NaCl in 1 liter of dH_2_O) at 34°C. Variants of this strain were constructed to contain the oRiboT and selection/screen constructs described below. All strain variants were grown under the same conditions with the exception of supplementation with inducers (aTc, IPTG, and arabinose) or antibiotics as described.

### Plasmid construction

Plasmids containing the oRiboT were derived from the RiboT-2 plasmid (*31*). The promoter controlling the expression of oRiboT was replaced with PL-tetO (*78*) such that transcription of the ribosome variants can be controlled by the TetR protein and aTc (Sigma-Aldrich). These plasmids were used as templates to construct all ribosome variants described in this study. The oEcoRI-oCAT-oGFP selection/screen plasmid was derived from plpp5-GFP (*31*) with the addition of EcoRI under the control of the arabinose–pBAD-inducible promoter and orthogonal ribosome binding site (RBS) (*31*, *33*) and CAT under the control of the aTc–pLtetO-inducible promoter and orthogonal RBS (*33*). All plasmids were assembled using Gibson assembly (NEB).

### Construction of oRiboT libraries

Libraries of randomized bases in the PTC with a theoretical diversity of ~1 × 10^3^ were constructed by randomizing 23*S* nucleotides 2058–2062, 2447–2451, 2495–2499, 2500–2504, and 2503–2507 (theoretical diversity of ~1 × 10^3^) and by randomizing nucleotides 2583–2588 (theoretical diversity of ~4 × 10^3^) using a QuikChange Lightning site-directed mutagenesis kit (Agilent). The libraries were transformed into EcNR1 cells containing the oEcoRI-oCAT-oGFP selection/screen plasmid in six separate transformations of the purified plasmid library and pooled. Transformations led to around 4× colony-forming units. This oversampling was necessary to be confident that the theoretical diversity of the library was maximally captured during transformations. Libraries were also verified by sequencing clones picked after transformation.

For validation of the determination of the fitness landscape of oRiboT in bases 2053–2058, Gibson assembly (NEB) was used in addition to QuikChange Lightning to create the 2053–2058 oRiboT plasmid libraries. Briefly, an oligonucleotide primer containing homology to the 23*S* ribosomal RNA (rRNA) of oRiboT and degenerate bases in region 2053–2058 in the 23*S* RNA of oRiboT and a reverse primer complementary to the 23*S* rRNA of oRiboT were used to amplify a region of oRiboT with homology to the backbone of the oRiboT plasmid and assembled with Gibson isothermal assembly. The purified plasmid library was then transformed into EcNR1 cells containing the oEcoRI-oCAT-oGFP selection/screen plasmid in six separate transformations and pooled.

### Negative selections

Ribosomal libraries in EcNR1 cells containing the oEcoRI-oCAT-oGFP selection/screen plasmid were grown in LB-min medium with induction of oEcoRI and oRiboT (+arabinose +aTc) and in kanamycin and carbenicillin for 16 hours. The surviving population was then grown to confluence and grown for 16 hours with induction of oGFP and oRiboT (+IPTG +aTc). The oGFP fluorescence of cells across the population was quantified with flow cytometry using the BD FACSAria, using BD FACSDiva v.8.0 software for acquisition. Cells with low oGFP fluorescence were then sorted using FACS into a negative bin to isolate cells with nonfunctional or impaired oRiboT mutants. All sorted cells were grown overnight, and confluent cultures were used to purify plasmids for NGS analysis. The FlowJo package was used for flow cytometry data analysis.

### Positive selections

Ribosomal libraries in EcNR1 cells containing the oEcoRI-oCAT-oGFP selection/screen plasmid were grown in LB-min medium with induction of oCATI and oRiboT (+aTc) and in kanamycin and carbenicillin for 16 hours. The surviving population was then grown to confluence and grown for 16 hours with induction of oGFP and oRiboT (+IPTG +aTc). The oGFP fluorescence of cells across the population was quantified with flow cytometry using BD FACSAria, using BD FACSDiva v.8.0 software for acquisition. Cells with high oGFP fluorescence were then sorted using FACS into a positive bin to isolate cells with functional oRiboT mutants. All sorted cells were grown overnight, and confluent cultures were used to purify plasmids for NGS analysis.

### NGS analysis to determine relative enrichment of oRiboT mutants

To create libraries for NGS, plasmid DNA of each of ~1 × 10^3^ or ~4 × 10^3^ cells in each oRiboT library was extracted using a Qiagen plasmid purification kit and polymerase chain reaction was used for targeted amplification of the sequencing region using barcoded primers corresponding to the positive selections, negative selections, and unselected populations. For the positive selections, barcodes ACTGAAGTACTATG and CGATATTCATGCTC were used for the forward and reverse primers, respectively. For the negative selection, barcodes TCAGGATGGACCAT and TCGACTGGGTGCAA were used for the forward and reverse primers, respectively. For the unselected population, barcodes TGTATCTAAGTAC and CCTACGTAGAGTT were used for the forward and reverse primers, respectively. Up to two libraries were pooled for sequencing using Illumina HiSeq.

Samples were pooled and sequenced on an Illumina HiSeq v2 Micro flowcell with 2 × 150–base pair paired-end reads. The .fastq files obtained from sequencing were analyzed with custom Python scripts. Briefly, after quality filtering, reads were searched for barcodes corresponding to the positive selection, negative selection, or unselected population. Next, reads were searched for the sequence adjacent to the site of mutagenesis for each library, and total WT and mutant reads were quantified. The total number of reads of each unique sequence in each library was quantified, and the degree of enrichment of each mutant sequence in each library was computed for both the positive and negative selections compared to the unselected population.

### Estimation of fitness of oRiboT mutants from relative enrichment after selections

We estimated the relative fitness of each oRiboT mutant by using the following equation

$$f(A)=\frac{A_{p}-A_{0}}{A_{n}-A_{0}}$$

where *f*(*A*) is the fitness of each oRiboT mutant, and the enrichment of each oRiboT mutant (determined by quantification of the NGS reads as detailed above) is given by *A*_p_ in the positive selection, *A*_n_ in the negative selection, and *A*_0_ in the unselected population. The raw fitness values of each library were plotted as a smoothed histogram to examine distribution of fitness values across all library members. As all libraries displayed a normal distribution of fitness values, the calculated fitness values of each library were normalized by the mean and SD so that fitness predictions of each library could be compared.

### Determination of the mutational flexibility of PTC bases

We computed the Shannon entropy (*44*) at each position to quantify the flexibility to mutation (*10*, *45*) at each site, using the following equation

$$H(X)=-\sum_{i=1}^{n}P(x_{i})\;\log P(x_{i})$$

where *H*(*X*) is the Shannon entropy and *P*(*x_i_*) is the probability of an A, C, U, or G at that position. Thus, *H*(*X*) has a minimum value of 0 when there is only a single base observed at each position, and a maximum value of 2 when there is an equal probability of observing any nucleotide at each position.

### Epistasis estimation from fitness values

On the basis of previous analysis of epistasis between the bases of an RNA element (*8*), we computed epistasis using the following equation

$$\varepsilon =f(AB_{i})-f(A_{i})\;\cdot \;f(B_{i})$$

where ε is the epistasis, *f*(*A*) is the fitness of point mutant with point mutation *A*, *f*(*B*) is the fitness of point mutant with point mutation *B*, and *f*(*AB*) is the fitness of a mutant having both mutations *A* and *B*. This measure of epistasis examines how, for every possible pair of point mutants, the combination of their mutations influences the fitness of a resultant dual mutant. Only mutant sequences having a point mutation or dual point mutation from the WT sequence were included in this analysis. We further normalized the epistasis values of each library by the mean and SD so that epistasis predictions could be compared between libraries.

## Notes

View/request a protocol for this paper from *Bio-protocol*.
