# Supplementary material for: Mapping the in vivo fitness landscape of a tethered ribosome
Source: Sci Adv. 2023 Apr 28;9(17):eade8934. doi: 10.1126/sciadv.ade8934 (PMC10146877; doi:10.1126/sciadv.ade8934)
Supplement: Supplementary file 1 — Table S1 Figs. S1 to S12 [file sciadv.ade8934_sm.pdf]

Science Advances
AAAS

Supplementary Materials for

Mapping the in vivo fitness landscape of a tethered ribosome

Felix Radford *et al.*

Corresponding author: Farren J. Isaacs, farren.isaacs@yale.edu

Sci. Adv. **9**, eade8934 (2023)
DOI: 10.1126/sciadv.ade8934

This PDF file includes:

Figs. S1 to S12
Table S1

Supplementary Tables

Low flexibility to mutation	A2060, A2062, A2450, G2495, U2500, C2507, U2584, and G2588
Medium flexibility to mutation	A2058, A2059, G2061, A2448, U2449, A2451, C2496, A2497, C2498, C2499, G2502, A2503, U2506, and U2585
High flexibility to mutation	G2447, C2501, G2505, G2583, U2586, and A2587

Table S1. Bases in the PTC and exit tunnel of oRiboT entrance exhibiting low, medium, and high flexibility to mutation.

Supplementary Figures

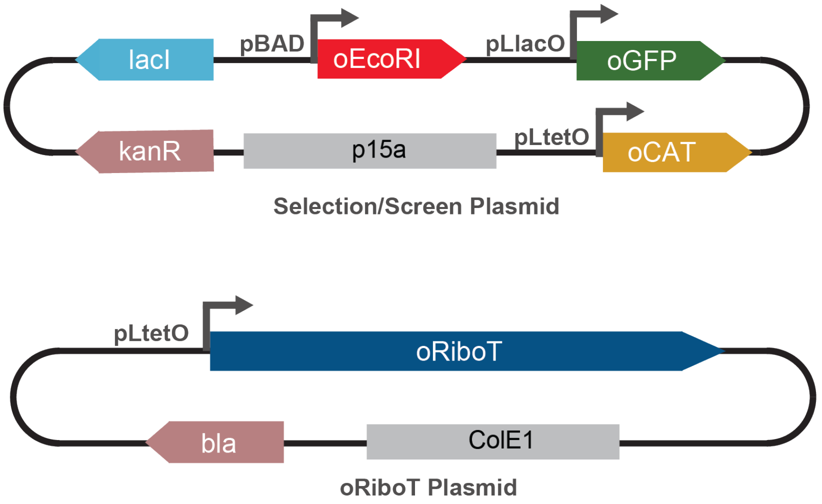

Fig. S1. The plasmids used for the selections and screens in this study.

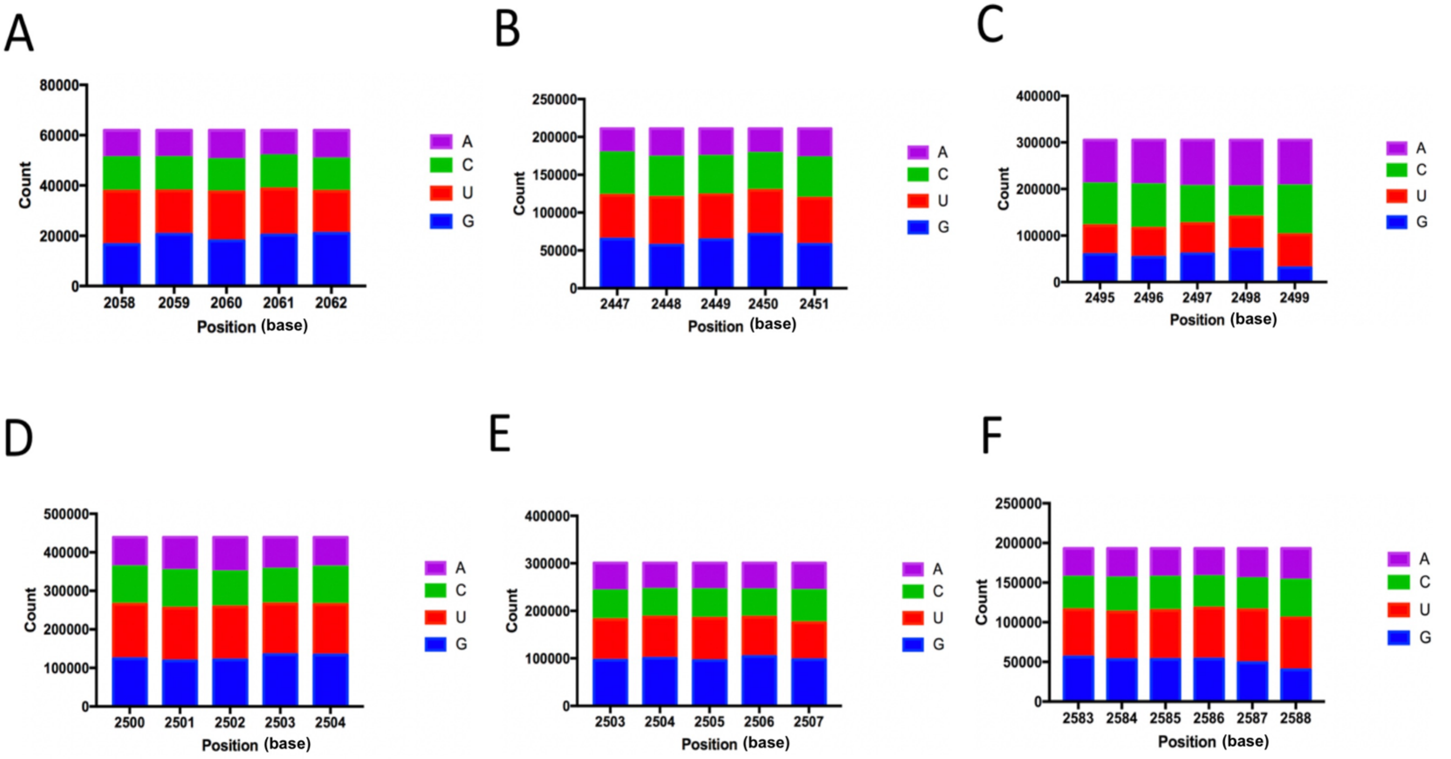

Fig. S2. Measurement of base frequencies at each position across the libraries studied.

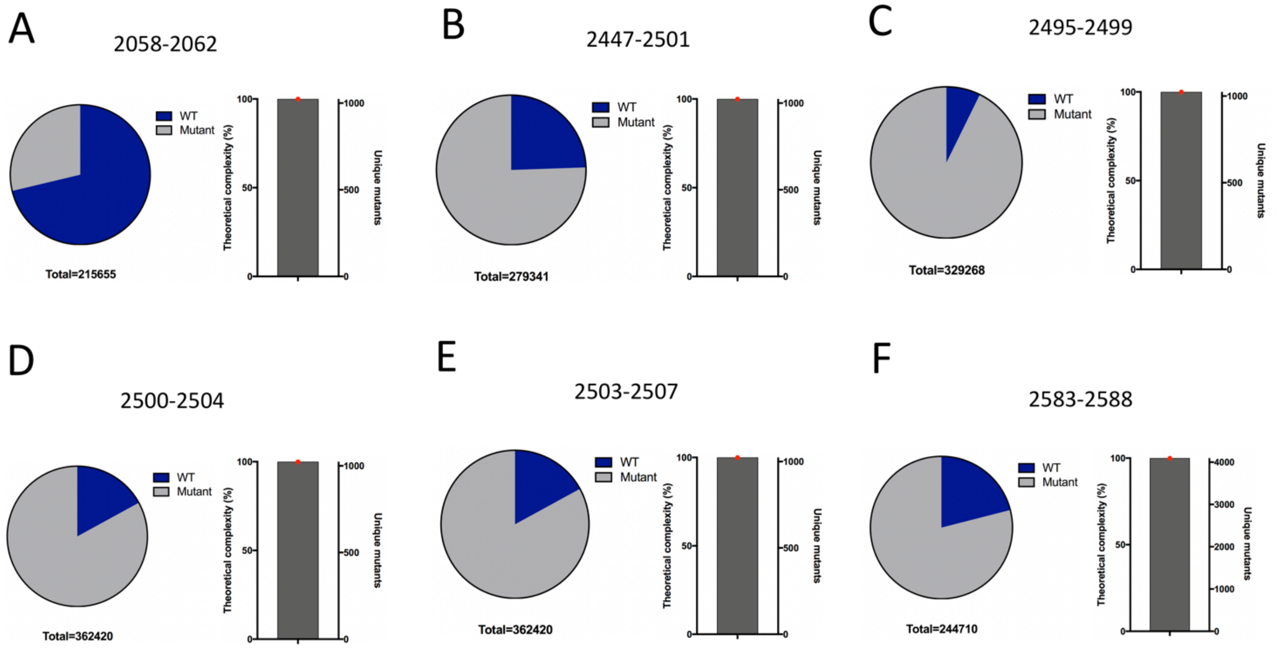

Fig. S3. Determination of theoretical complexity achieved in each library and percentage of WT sequences relative to mutants.

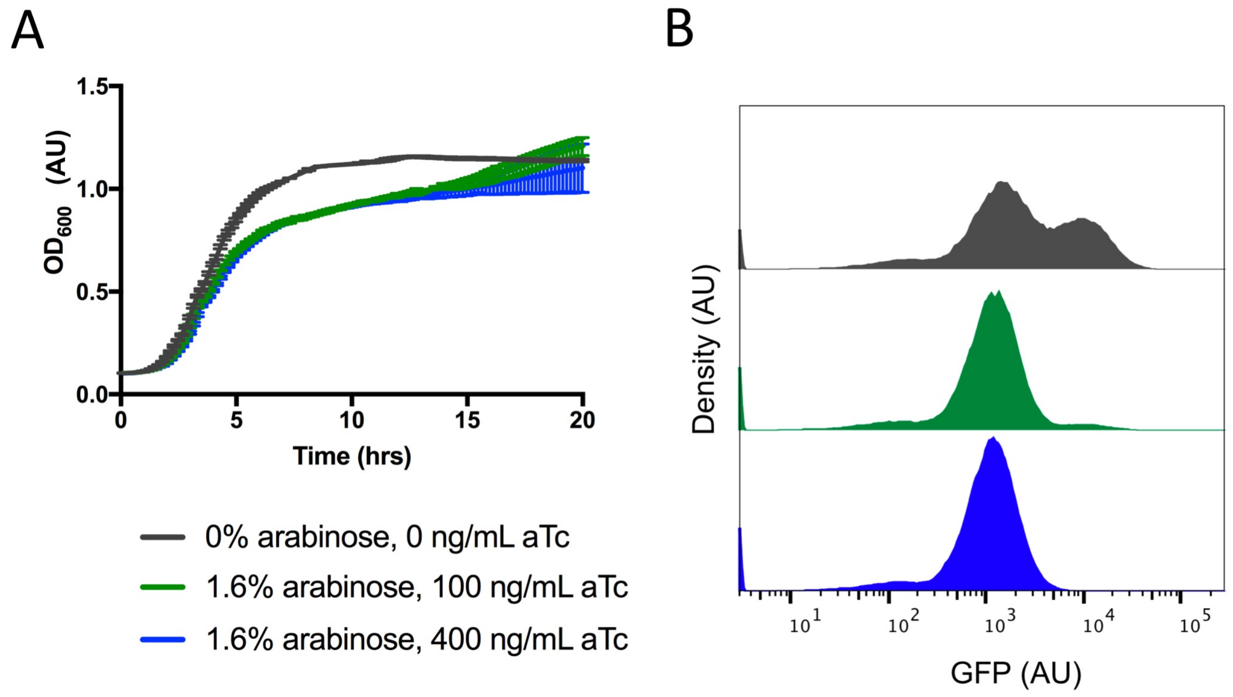

Fig. S4. Conditions for oEcoRI selections. The conditions for the oEcoRI selection were varied to determine the optimal inducer concentration. (A) Growth curves in each inducer set. Each of these conditions were subsequently induced with IPTG and aTc in order to express oGFP and (B) oGFP induction was measured with flow cytometry.

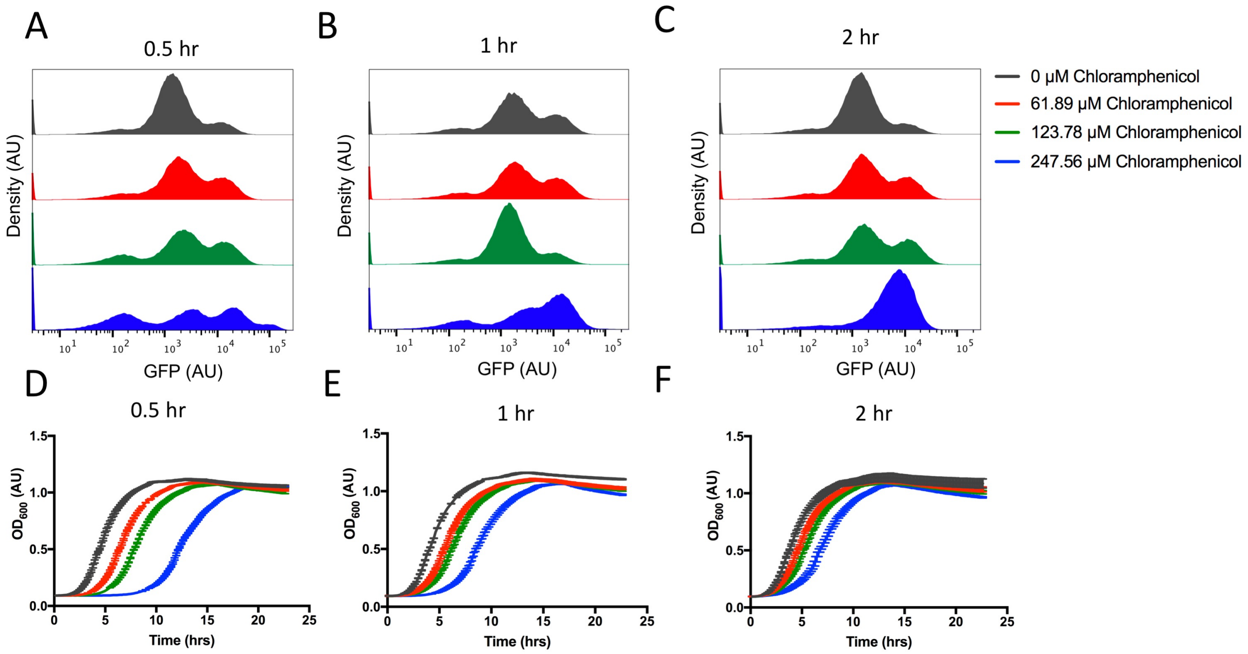

Fig. S5. Determining optimal conditions for oCAT selection. (A) 0.5 hr induction flow cytometry after induction of oGFP, (B) 1 hr induction flow cytometry after induction of oGFP, (C) 2 hr induction flow cytometry after induction of oGFP, (D) growth curves in chloramphenicol after 0.5 hr aTc induction, (E) growth curves in chloramphenicol after 1 hr aTc induction, and (F) growth curves in chloramphenicol after 2 hr aTc induction.

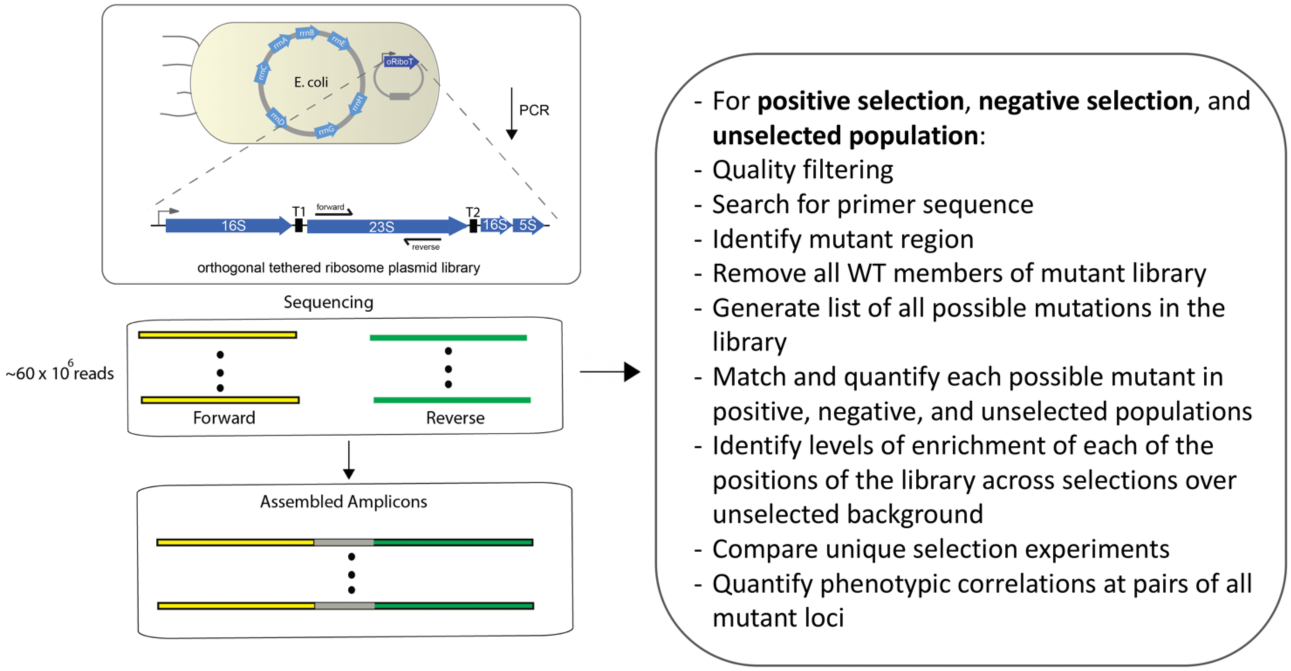

Fig. S6. The next generation sequencing workflow used to determine the fitness landscape of oRiboT. The plasmid libraries from independent positive and negative selections as well as the un-selected populations were extracted and amplified with PCR. Illumina sequencing was performed and the levels of enrichments in each condition were used to reconstruct the fitness of each mutant in the population and construct a fitness landscape of oRiboT (**See Materials and Methods** for further details).

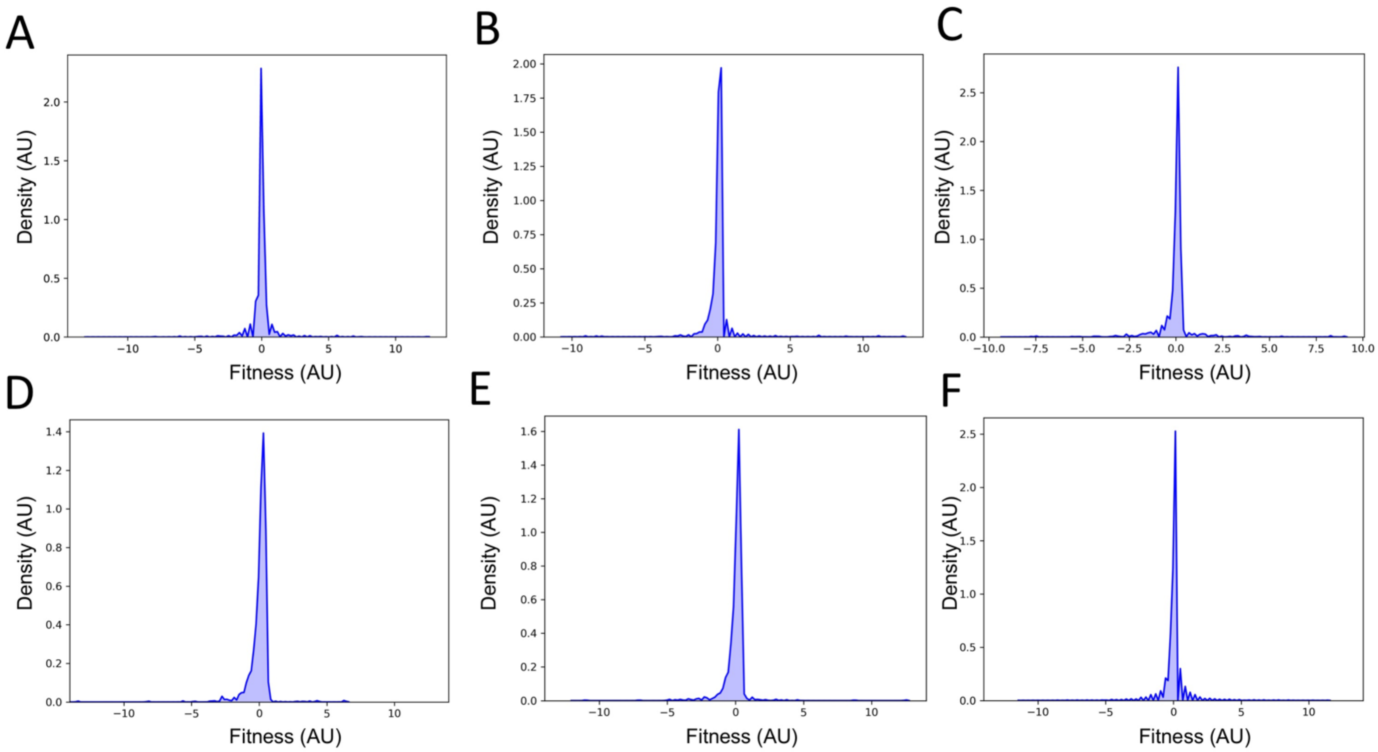

Fig. S7. Distributions of fitness of sequences in each library. (A) 2058-2062 library, (B) 2447-2451 library, (C) 2495-2499 library, (D) 2500-504 library, (E) 2503-2507 library, and (F) 2583-2588 library.

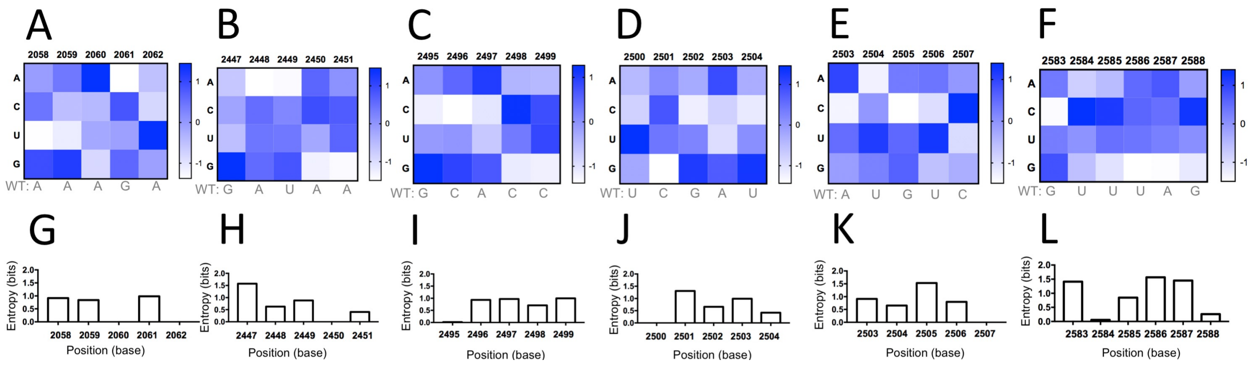

Fig. S8. **Enrichments and Shannon entropies for mutation for all of the libraries profiled in this study.** Enrichment matrices for the (A) 2058-2062 library, (B) 2447-2451 library, (C) 2495-2499 library, (D) 2500-504 library, (E) 2503-2507 library, and (F) 2583-2588 library. Entropies for the (G) 2058-2062 library, (H) 2447-2451 library, (I) 2495-2499 library, (J) 2500-504 library, (K) 2503-2507 library, and (L) 2583-2588 library. An entropy of 0 signifies low flexibility to mutation, whereas an entropy of 2 is a very high flexibility to mutation, implying equal probability of obtaining any of the four bases at that position.

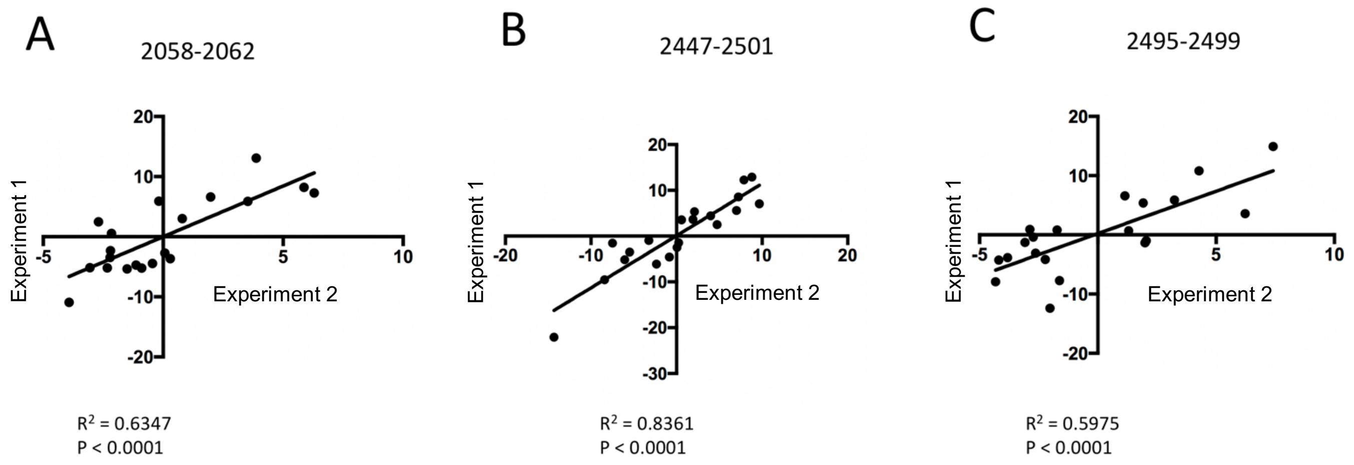

Fig. S9. Reproducibility between independent selection performed on each library. The enrichment of bases at each position of libraries (A) 2058 library, (B), 2447-2051 library, and (C) 2495-2499, after positive and negative selections was determined (as in **Fig. S8A-F**) for two independent sets of selection experiments. The reproducibility and significance of the base enrichments of each of two independent selection experiments were compared for each library.

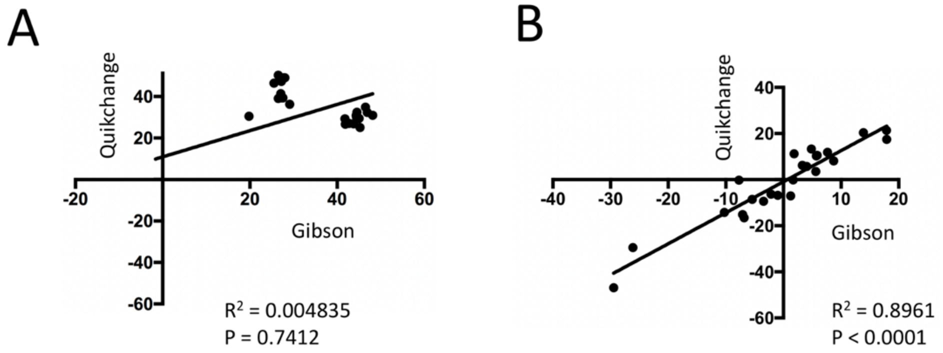

Fig. S10. Comparison of independent preparations of the 2583-2588 library before and after our selection methodology. (A) The initial frequencies of bases at each position in the 2583-2588 libraries constructed with Gibson isothermal assembly or Quikchange mutagenesis, respectively. (B) The frequencies of bases at each position in the 2583-2588 libraries post-selections.

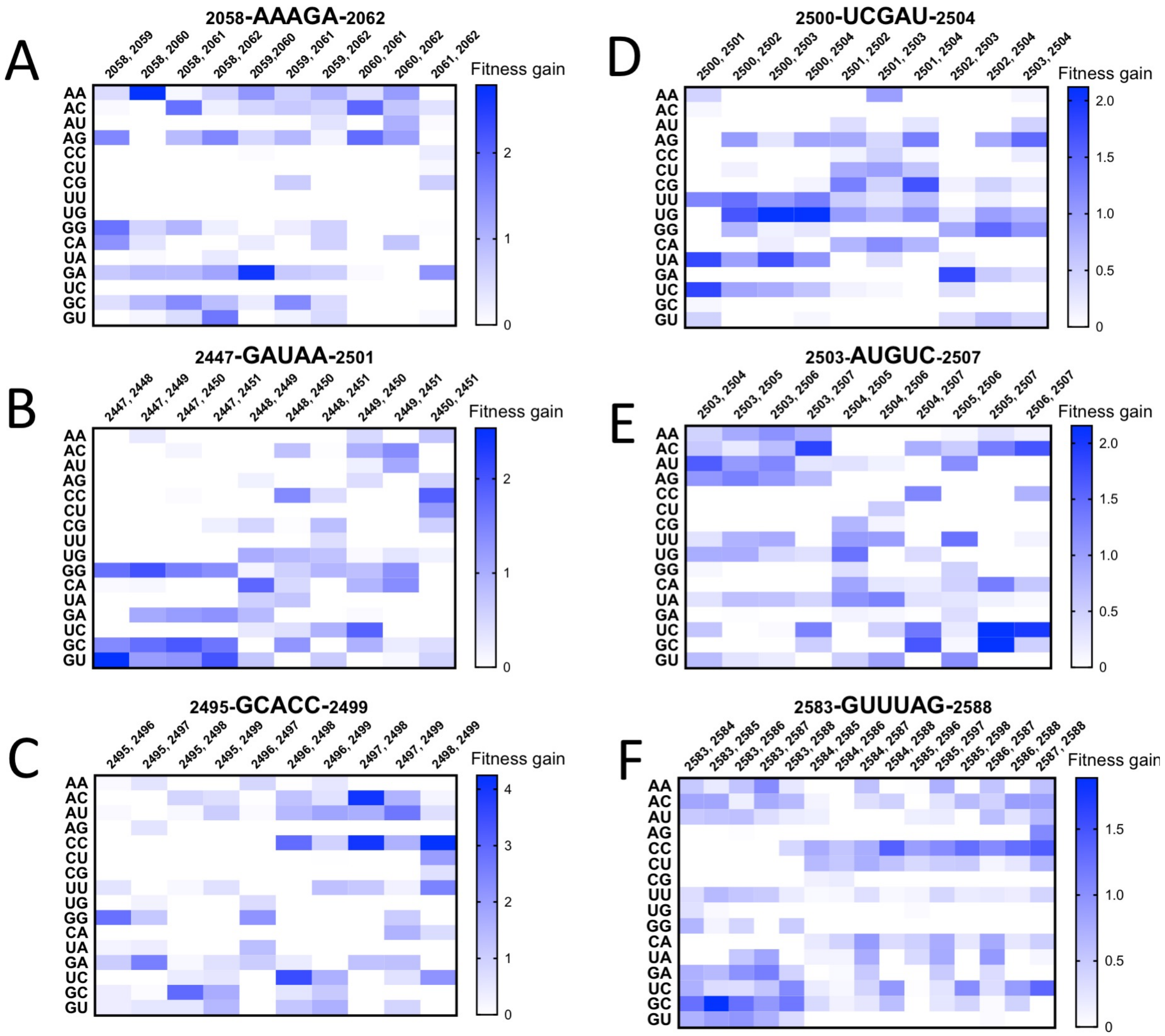

Fig. S11. The covariation of all base pairs between all pairs of positions in each of the oRiboT libraries profiled. We examined all possible combinations of two positions in each library and calculated their effect on the fitness **(Materials and Methods)** of oRiboT. Heat maps represent the fitness gain from the enrichment at each pair of nucleobase combinations.

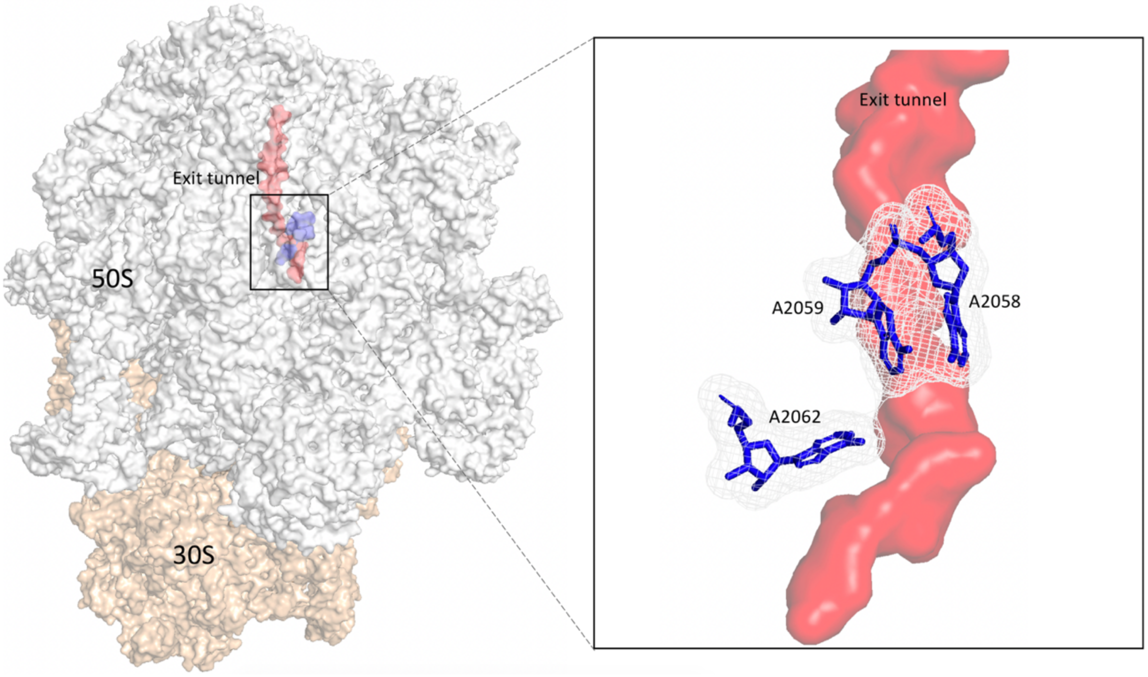

Fig. S12. The bases 2058, 2059, and 2062 form a critical "chokepoint" in the exit tunnel.
